# RNA-Seq Provides Novel Genomic Resources for Noug (*Guizotia abyssinica*) and Reveals Microsatellite Frequency and Distribution in Its Transcriptome

**DOI:** 10.3389/fpls.2022.882136

**Published:** 2022-05-11

**Authors:** Adane Gebeyehu, Cecilia Hammenhag, Kassahun Tesfaye, Ramesh R. Vetukuri, Rodomiro Ortiz, Mulatu Geleta

**Affiliations:** ^1^Department of Plant Breeding, Swedish University of Agricultural Sciences, Lomma, Sweden; ^2^Ethiopian Biotechnology Institute, Addis Ababa, Ethiopia; ^3^Institute of Biotechnology, Addis Ababa University, Addis Ababa, Ethiopia

**Keywords:** *de novo* transcriptome assembly, G+C content, genetic variation, self-compatibility, SNPs, SSR, unigenes

## Abstract

Genomic resources and tools are essential for improving crops and conserving their genetic resources. *Guizotia abyssinica* (noug), an outcrossing edible oilseed crop, has highly limited genomic resources. Hence, RNA-Seq based transcriptome sequencing of 30 noug genotypes was performed to generate novel genomic resources and assess their usefulness. The genotypes include self-compatible and self-incompatible types, which differ in maturity time, photoperiod sensitivity, or oil content and quality. RNA-Seq was performed on Illumina HiSeq 2500 platform, and the transcript was reconstructed *de novo*, resulting in 409,309 unigenes. The unigenes were characterized for simple sequence repeats (SSRs), and served as a reference for single nucleotide polymorphism (SNP) calling. In total, 40,776 SSRs were identified in 35,639 of the 409,309 unigenes. Of these, mono, di, tri, tetra, penta and hexanucleotide repeats accounted for 55.4, 20.8, 21.1, 2.3, 0.2, and 0.2%, respectively. The average G+C content of the unigenes and their SSRs were 40 and 22.1%, respectively. The vast majority of mononucleotide repeat SSRs (97%) were of the A/T type. AG/CT and CCA/TGG were the most frequent di and trinucleotide repeat SSRs. A different number of single nucleotide polymorphism (SNP) loci were discovered in each genotype, of which 1,687 were common to all 30 genotypes and 5,531 to 28 of them. The mean observed heterozygosity of the 5,531 SNPs was 0.22; 19.4% of them had polymorphism information content above 0.30 while 17.2% deviated significantly from Hardy-Weinberg equilibrium (*P* < 0.05). In both cluster and principal coordinate analyses, the genotypes were grouped into four major clusters. In terms of population structure, the genotypes are best represented by three genetic populations, with significant admixture within each. Genetic similarity between self-compatible genotypes was higher, due to the narrow genetic basis, than that between self-incompatible genotypes. The genotypes that shared desirable characteristics, such as early maturity, and high oil content were found to be genetically diverse, and hence superior cultivars with multiple desirable traits can be developed through crossbreeding. The genomic resources developed in this study are vital for advancing research in noug, such as genetic linkage mapping and genome-wide association studies, which could lead to genomic-led breeding.

## Introduction

Noug (*Guizotia abyssinica*) is an edible oilseed crop indigenous to Ethiopia, where it was originated, domesticated and genetically diversified. It is an annual diploid crop with 2n = 30 chromosomes (Dagne, 1994) exhibiting a strict outcrossing reproductive mechanism with honeybees as major pollinators due to its homomorphic self-incompatibility (Geleta et al., 2002; Geleta, 2007; Geleta and Bryngelsson, 2010). It is among major edible oilseed crops grown in Ethiopia, both in terms of acreage and production volume, where 26% of the produce is consumed locally (Geleta and Ortiz, 2013; Ethiopian Institute of Agricultural Research [EIAR], 2017). It is also cultivated to some extent in other African countries that include Sudan, Malawi and Uganda (Geleta and Ortiz, 2013; Gebeyehu et al., 2021). Apart from Africa, it is cultivated in India as a minor oilseed crop, as well as in Bangladesh, the Caribbean, and the United States, however to a much lesser extent (Geleta and Ortiz, 2013).

Genetic diversity in crops refers to the genetic variation within and between individual plants, landrace populations, and cultivars, which results from mutation, recombination, introgression, natural and artificial selection, and adaptation to diverse environments. A crop’s genetic diversity is typically greatest in areas where it was domesticated, originated, or has wild relatives (Geleta and Ortiz, 2013). This diversity plays a key role in the crop’s ability to adapt to climate change and withstand new pests, as well as to increase its productivity and quality. Since Ethiopia is its center of origin and diversity, noug cultivated in the country is inherently diverse with high genetic potential for improvement (Geleta et al., 2007, 2008; Petros et al., 2007; Dempewolf et al., 2010; Mengistu et al., 2020; Tsehay et al., 2020). However, the genetic potential of this crop has not been widely exploited, and only a few modestly improved cultivars have been released (Alemaw and Alamayehu, 1997). Among the major constraints are strict self-incompatibility, which requires abundant availability of insect pollinators, an indeterminate growth habit that leads to seed loss due to shattering, lodging, low response to management and inputs, and pests (including various pathogens, insects and parasitic weeds).

The process of cultivar development for a crop begins with selecting genetic material with desirable traits. For efficient selection of genetic material for breeding, understanding the genetic variation within a crop’s gene pool is vitally important using DNA markers. Thus, it is imperative that genome-wide markers be developed and utilized in order to identify and manage genetic diversity within a crop’s gene pool and to determine genetic factors determining desirable traits. To interpret the functional elements of a genome, it is essential to understand its transcriptome, which include sequence variation in their mRNA transcripts (Wang et al., 2009). As transcriptome markers represent the expressed parts of a genome, they are a better choice than genomic markers for aforementioned applications. To this end, a limited number of transcriptome sequences have been assembled for noug (Dempewolf et al., 2010; Hodgins et al., 2014; Tsehay et al., 2020), and based on these, simple sequence repeat (SSR) markers and single nucleotide polymorphism (SNP) markers have been developed (Dempewolf et al., 2010; Tsehay et al., 2020). However, these genomic resources are insufficient for use in different applications including population genetics analyses for conservation; genome-wide association studies (GWAS) as well as for enabling genomics-led breeding. Hence, the development of additional genomic resources for noug is vitally important.

RNA-Seq (RNA sequencing) is the most advanced method of profiling transcriptomes, which relies on next-generation sequencing methods for high-throughput (Wang et al., 2009). The capability of detecting sequence variations, such as Indels and SNPs in transcribed genomic regions are among the key advantages of RNA-Seq (Cloonan et al., 2008). Additionally, the unigenes obtained after transcriptome assembly can be used in the development of other markers, such as SSRs. The aims of this study were to use RNA-Seq for transcriptome sequencing of diverse genotypes of noug for the development of new genomic resources for their various applications, characterize the SSRs in the unigenes, and assess the usefulness of the novel SNP markers *via* genetic diversity analyses of the genotypes used.

## Materials and Methods

### Plant Material

Thirty phenotypically diverse noug genotypes were used in this study (Supplementary Table 1). Most of the genotypes were selected from breeding populations bred for desirable traits such as self-compatibility, early maturity, less-sensitivity to photoperiod, as well as high oil or increased oleic acid contents (Geleta and Bryngelsson, 2010; Geleta et al., 2011; Geleta and Ortiz, 2013). Other genotypes were selected from landrace populations based on their distinct differences in one or more traits from those that were already selected (Supplementary Table 1). Twelve of the 30 genotypes are self-compatible although to a different extent, whereas the remaining eighteen are strictly self-incompatible. In terms of maturity time, the source populations varied from very-early to very-late types. For three of the 30 genotypes, the source populations were able to flower when the photoperiod was above 12 h. The average oil content of the source populations varied from 30 to 45% of dry seed weight. As opposed to the other source populations, four have oleic acid content above 10%, although the level depends primarily on environmental temperature (Supplementary Table 1).

### Planting, Sampling and RNA Extraction

The 30 genotypes were planted using 1.5 L plastic pots filled with soil in a greenhouse at the Swedish University of Agricultural Sciences (SLU, Alnarp, Sweden) for RNA extraction. Four weeks after planting, leaf tissue was collected separately from individual plants of each genotype in 15 ml falcon tubes and snap-frozen in liquid nitrogen and then stored at −80°C until used for RNA extraction. For each sample, the total RNA was extracted from approximately 100 mg leaf tissue using the RNeasy Plant Mini Kit (#74904, QIAGEN) according to the manufacturer’s protocol, followed by DNase treatment with Ambion Turbo DNA-Free Kit (#AM1907, Thermo Fisher Scientific, CA, United States) as described in Kalyandurg et al. (2021). The extracted RNA quality and quantity were assessed using an Agilent Bioanalyzer 2100 system (Agilent, Technologies, CA, United States), NanoDrop ND-1000 spectrophotometer (Saveen Werner, Sweden), and agarose gel electrophoresis. Then, high-quality RNA samples were sent to CD Genomics (New York, United States) for RNA-Seq analysis. Upon arrival, the samples were further monitored on 1% agarose gels for degradation and contamination, purity checked using the NanoPhotometer spectrophotometer (IMPLEN, CA, United States), concentration measured using the Qubit RNA Assay Kit in Qubit 2.0 Flurometer (Life Technologies, CA, United States), integrity assessed using the RNA Nano 6000 Assay Kit of the Agilent Bioanalyzer 2100 system (Agilent Technologies, CA, United States).

### Library Preparation, Clustering and Sequencing

The NEBNext UltraTM RNA Library Prep Kit for Illumina (NEB, United States) was used to create sequencing libraries from 1.5 μg of RNA per sample, according to the manufacturer’s instructions, and index codes were added to assign sequences to each sample. An AMPure XP system (Beckman Coulter, Beverly, United States) was used to purify the library fragments to facilitate preferential selection of cDNA fragments with a length of 150–200 bp. Following adapter ligation to the size-selected fragments and polymerase chain reaction (PCR), the AMPure XP system was used to purify the amplified products, and then library quality was assessed using the Agilent Bioanalyzer 2100 system. This was followed by the clustering of the index-coded samples on a cBot Cluster Generation System using the TruSeq PE Cluster Kit v3-cBot-HS (Illumia) as per the manufacturer’s instructions. The clusters were then sequenced on the Illumina HiSeq 2500 platform, and paired-end reads were generated.

### Data Quality Control, *de novo* Transcript Assembly and Splicing, and SSR Identification

The Illumina Hiseq data was translated to sequenced reads through base calling, and a FASTQ file containing sequenced reads and quality information was created from the raw data for each sample. A series of methods was applied to filter the raw sequencing reads to obtain high quality data for subsequent analysis. First, the raw reads in FASTQ format were processed using in-house python scripts, and reads containing adapter and ploy-N were removed to obtain clean reads. The Phred quality scores of the clean reads were then calculated, and those with Phered quality scores below 30 (error rate greater than 0.1%) were removed. The remaining high-quality reads were used for downstream analyses.

Since noug does not have a reference genome, *de novo* transcript reconstruction was done using Trinity software package (Grabherr et al., 2011). For this, read1 files containing high-quality reads for each of the 30 samples were merged into a single read1 file, and similarly the read2 files of the 30 samples were merged into a single read2 file. The merged read1 and read2 files were then used for transcript assembly and splicing using Trinity, by setting max_kmer_cov to 2 and all other parameters to default. Following length distribution analysis, the longest spliced transcript for each gene was identified as a unigene and used as a reference sequence in subsequent analyses. This resulted in 409,309 unigenes with a G+C content of 40%, which were used as reference for SNP calling. A web-based microsatellite identification tool MISA-web (Beier et al., 2017^1^) was used to identify simple sequence repeats (SSRs) within the unigenes using the default setting. The minimum number of repeats was set to ten for mononucleotide repeats, to six for dinucleotide repeats and to five for tri, tetra, penta and hexanucleotide repeats.

### SNP Calling and Further Processing

As the first step of SNP calling, the BWA v.0.7.4 short read aligner was used to align the high-quality clean reads of each sample to the reference transcripts (Li and Durbin, 2009). Then, SAMtools v0.1.18 (Li et al., 2009) and Picard-tools v1.41 software packages were used for sorting, indexing, removing duplicates, and merging the BAM alignment results of each sample. On the merged BAM files, the Genome Analysis Toolkit (GATK; McKenna et al., 2010) was used for base-quality score calibration, and SNP calling, and genotyping for each sample was performed by using standard filtering parameters or variant quality score calibration according to GATK’s Best Practice recommendations (DePristo et al., 2011; Van der Auwera and O’Connor, 2020). The VCF files of the samples were then merged and the shared SNP loci were filtered using BCFtools (Danecek et al., 2021).

### Statistical Analysis

Different statistical programs were used to estimate genetic diversity parameters and indices for each genotype across loci and for each locus across genotypes. GenAlEx version 6.5 software (Peakall and Smouse, 2006) was used for the analysis of mean values of observed number of alleles (Na), observed heterozygosity (Ho), number of private alleles (NPA), percent polymorphic loci (PPL) for each genotype, Nei’s standard genetic distance (GD) and GD-based principal coordinate analysis (PCoA) to display the genetic relationship between the noug genotypes based on both SNP data sets. Pairwise GD matrices were also used for neighbor joining (NJ)-based cluster analysis using the MEGA7 program (Kumar et al., 2016). The polymorphism information content of each SNP locus was calculated in accordance with Hildebrand et al. (1992). Arlequin v. 3.5.2.2 (Excoffier and Lischer, 2010) was used to perform the exact test of Hardy-Weinberg equilibrium (using 1,000,000 steps in the Markov chain and 100,000 dememorization steps), and calculate pairwise F_ST_ and mean number of pairwise differences between and within genotypes and groups. To generate heatmaps of these parameters, a console version of the R statistical package (Rcmd) incorporated into the Arlequin software was used. A Bayesian statistics based population genetic structure analysis was conducted using STRUCTURE software version 2.3.4 (Pritchard et al., 2000). The analysis was conducted using an admixture model for different number of clusters (K) using 100,000 burn-in periods and 200,000 Markov chain Monte Carlo (MCMC) chain iterations, with K ranging from two to ten and twenty replications at each K. A further analysis of the results was performed with the STRUCTURESELECTOR (Li and Liu, 2018) program to determine the number of clusters (genetic populations) according to the Puechmaille (2016) method, and to visualize the population structure using CLUMPAK (Kopelman et al., 2015) integrated into STRUCTURESELECTOR.

## Results

### SSR Identification and Characterization

**FIGURE 1:**
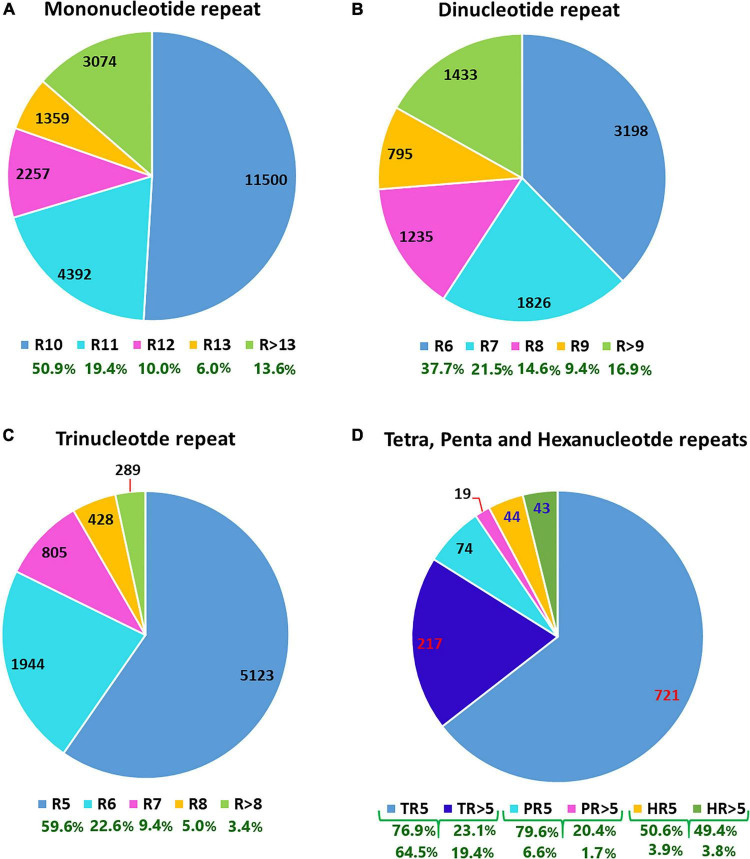
P-charts depicting the number (values in the pie charts) and percentage (values shown below the chart keys in green) of different simple sequence repeat motifs across the noug unigenes that were classified based on the number of times they were repeated. In the chart keys of panels **(A–C)**, the numbers following “R” denote the number of times the corresponding repeat motifs were repeated. In the chart keys of **(D)**, T, P and H refer to Tetra, Penta and Hexanucleotide repeats. “>” indicates that the SSR motif was repeated more times than the specified number.

**FIGURE 2:**
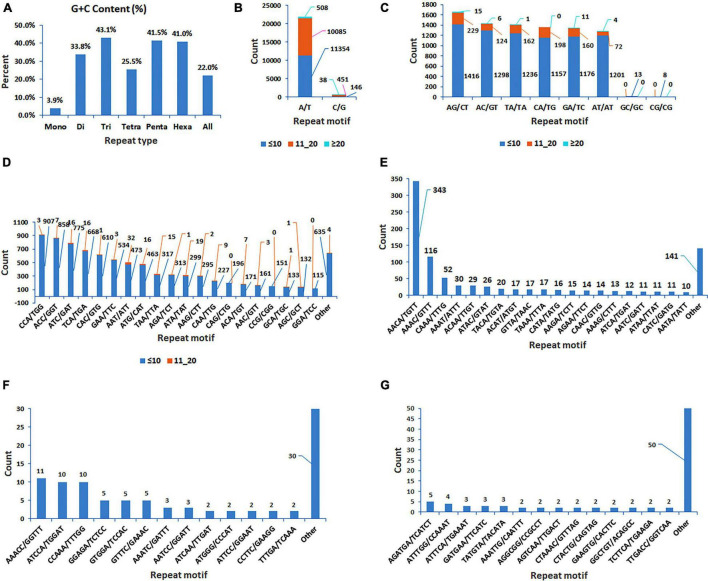
Bar graphs displaying the G+C content of mono, di, tri, tetra, penta and hexanucleotide repeat SSRs, and all SSRs together **(A)**; and the number of classified repeat types considering sequence complementarity) for mono, di, tri, tetra, penta and hexanucleotide repeat SSRs, which were grouped based on the number of times a repeat motif was repeated (three groups for mono and dinucleotide repeats, two groups for trinucleotide repeats, and one group for each of tetra, penta and hexanucleotide repeats) **(B–G)**.

The analysis of 409,309 unigenes using MISA-web for detecting SSRs resulted in 40,776 SSRs (Table 1). These SSRs were detected in 35,639 unigenes (8.7% the total unigenes), of which 4,269 had more than one SSR (1% of the total unigenes, or 12% of the unigenes containing SSRs). Some of these SSRs were separated by less than 100 bases and hence formed compound SSRs. Counting SSRs forming a compound SSR as one, the total number of SSRs was 38,011, of which 2,380 were compound SSRs (Table 1 and Supplementary Table 2). Among the 40,776 separate SSRs identified, mono-, di-, tri-, tetra-, penta- and hexanucleotide repeats accounted for 55.4, 20.8, 21.1, 2.3, 0.2, and 0.2%, respectively (Table 1 and Figure 1). In all cases, the lowest number of repeats accounted for the highest proportion. Among the mononucleotide repeat SSRs, 50.9% had a repeat of ten whereas 37.7% of dinucleotide repeat SSRs had a repeat of six. In the case of tri, tetra, penta and hexanucleotide repeats, a repeat of five accounted for 59.6, 76.9, 79.6, and 50.6%, respectively (Figure 1). In general, the longer a given SSR motif gets, the less frequent it becomes. The G+C contents of mono, di, tri, tetra, penta and hexanucleotide SSRs were 3.9, 33.3, 43.1, 25.5, 41.5, and 41.0%, respectively. Whereas, all SSRs together had a G+C content of 22.2% (Figure 2A).

**TABLE 1:** Summary information about the simple sequence repeat (SSR) analysis.

SSR Analysis	No. of genes	Percentage (%)
Total number of sequences examined (TNSE)	409,309	100^a^
Total size of examined sequences (bp)	204,196,448	
Number of SSR containing sequences	35,639	8.7^a^
Number of sequences containing more than one SSRs	4,269	1.0^a^
Total number of identified SSRs (TNIS)	40,776	100^b^
Number of mononucleotide repeat SSRs	22,582	55.4^b^
Number of dinucleotide repeat SSRs	8,487	20.8^b^
Number of trinucleotide repeat SSRs	8,589	21.1^b^
Number of tetranucleotide repeat SSRs	938	2.3^b^
Number of pentanucleotide repeat SSRs	93	0.2^b^
Number of hexanucleotide repeat SSRs	87	0.2^b^
Total number of SSRs (TNS)*	38,011	100^c^
Number of SSRs present in compound formation	2,380	6.3^c^

*Number of repeats considered for mononucleotide SSRs: ≥ 10.*

*Number of repeats considered for dinucleotide SSRs: ≥ 6.*

*Number of repeats considered for tri, tetra, penta and hexanucleotide repeats: ≥ 5.*

*Maximal number of bases interrupting two SSRs in a compound SSR: = 100.*

*Compound SSRs were counted as single SSRs unlike the case of TNIS.

*^a^%age of TNSE; _b_ = %age of TNIS; ^c^ = %age of TNS.*

The SSRs were further analyzed considering sequence complementarity (Figure 2). Among the mononucleotide repeat SSRs, the vast majority (97.2%) were A/T type whereas C/G type accounted for only 2.8% (Figure 2). The most and least common dinucleotide repeat SSRs were AG/CT (19.6%) and CG/CG (0.09%), respectively. CCA/TGG, ACC/GGT, and ATC/GAT were the top three most common trinucleotide repeat SSRs, accounting for 10.6, 10.1, and 9.2% of the total trinucleotide repeat SSRs, respectively. Among the tetranucleotide repeat SSRs, AACA/TGTT was by far the most frequent (36.7%), followed by AAAC/GTTT (12.4%). The most common pentanucleotide repeats were AAACC/GGTTT, ATCCA/TGGAT, and CCAAA/TTTGG (12, 11, and 11%, respectively). The frequency of different types of hexanucleotide repeats ranged from one to five, with AGATGA/TCATCT being the most common (Figure 2).

### The SNP Markers

**FIGURE 3:**
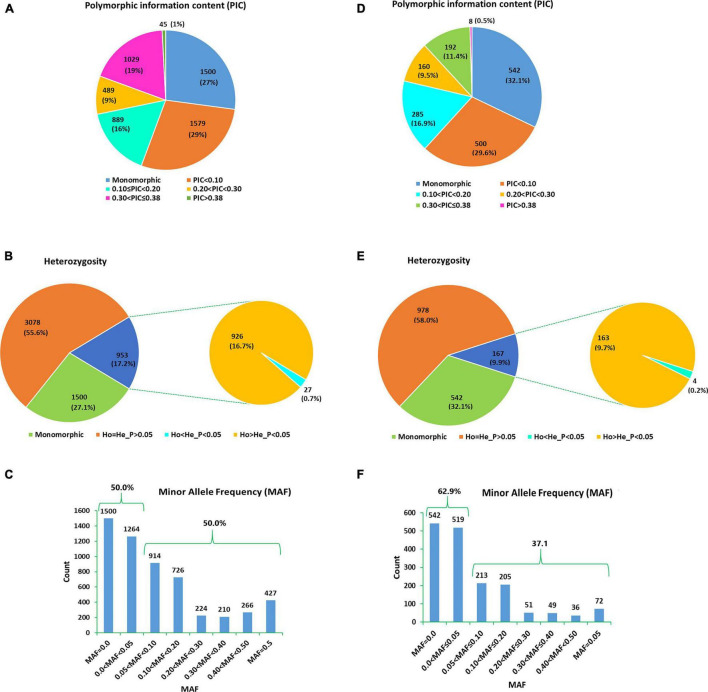
Pie charts/bar graphs exhibiting the grouping of 5,531 SNP loci recorded across the 28 noug genotypes according to **(A)** their polymorphism information content (PIC), **(B)** heterozygosity, and **(C)** minor allele frequency (MAF); and the 1,687 SNP loci recorded across the 30 noug genotypes according to **(D)** their PIC, **(E)** heterozygosity, and **(F)** MAF.

The number of high-quality SNPs discovered in each sample that met all filtering criteria ranged from 80,653 (in genotype Ga02.02) to 334,828 (in genotype Ga09.03) (Data not shown). Among the SNPs discovered in each genotype, 1,687 of them were shared among the 30 genotypes. In comparison, excluding two of the samples (Ga02.02 and Ga101B.m) that shared the least number of SNP loci with the others resulted in 5,531 SNP loci shared by the 28 remaining samples (Figure 3 and Supplementary Table 3). Both SNP datasets were used for further analyses and the results were compared. Out of the 5,531 SNP loci, 1,500 (27%) were monomorphic across the 28 genotypes whereas 542 of the 1,687 SNP loci (32.1%) were monomorphic across the 30 genotypes (Figure 3). Thus, the number of polymorphic SNPs was 4,031 for the 28 genotypes and 1,145 for the 30 genotypes.

Among the SNP loci shared by the 28 and 30 genotypes, 1,074 (19.4%) and 200 (11.9%) loci had a polymorphism information content (PIC) of above 0.30, respectively (Figure 3 and Supplementary Table 3), and hence are highly informative. Under the assumption that the genotypes constitute a random sample of a single population, the HWE test revealed that 953 loci (17.2% of the 5,531 loci) and 167 loci (9.9% of the 1,687 loci) showed significant deviation from HWE (*P* < 0.05) when the population comprised the 28 and 30 genotypes, respectively (Figure 3 and Supplementary Table 4). Among the 5,531 and 1,687 loci analyzed, 0.5 and 0.2% showed heterozygote deficiency, respectively (Figure 3). In total, 28 SNP loci across 27 unigenes exhibited heterozygote deficiency.

### Genetic Variation Within and Among Genotypes and Groups

For the genetic diversity analyses, the 5,531 and 1,687 SNPs were used for the two groups comprising 28 and 30 genotypes, respectively (Table 2). Among the 5,531 and 1,687 SNP loci, 50 and 37.1% had minor allele frequency (MAF) above 0.05 (Figures 3C,F). The analysis using the 5,531 SNPs resulted in observed heterozygosity (Ho) ranging from 0.18 (in genotype Ga01.12) to 0.28 (in genotype Ga08.05), which are the same as the percent polymorphic loci (PPL) of the genotypes. The overall mean observed number of alleles (Na) and Ho were 1.22 and 0.22, respectively. The average genetic distance (GD) of a genotype from the other genotypes ranged from 0.040 (genotype Ga01.20) to 0.055 (genotype Ga10.06), with an overall average of 0.048. Private alleles were detected in 82.1% of the 28 genotypes, with genotype Ga09.03 having the highest number of private alleles (NPA; mean = 0.014). The corresponding analysis using the 1,687 SNPs across the 30 genotypes produced Ho ranging from 0.12 (in genotypes Ga01.12 and Ga01.20) to 0.23 (in genotype Ga08.05) with an overall mean of 0.18 (Table 2). Whereas, an individual genotype’s GD from other genotypes ranged from 0.035 (Ga01.12) to 0.051 (Ga10.06), with an overall average of 0.043. In this group, private alleles were detected in all genotypes except in Ga01.12 and Ga01.16 (Table 2).

**TABLE 2:** Mean values of observed number of alleles (Na), observed heterozygosity (Ho), number of private alleles (NPA), percent polymorphic loci (%PL) for each genotype and Nei’s standard genetic distance (GD) of each genotype from all other genotypes based on data from 5,531 SNP loci (for 28 of the 30 genotypes) and 1,687 loci (for all 30 genotypes).

Genotype	28 genotypes and 5,531 Loci					30 genotypes and 1,687 loci				
	Mean Na ± SE	Mean Ho ± SE	Mean NPA ± SE	%PL	GD	Mean Na ± SE	Mean Ho ± SE	Mean NPA ± SE	%PL	GD
Ga01.12	1.18 ± d	0.18 ± d	0.001 ± a	0.18	0.041	1.12 ± f	0.12 ± f	0.000 ± a	0.12	0.035
Ga01.16	1.20 ± d	0.20 ± d	0.001 ± a	0.20	0.041	1.13 ± f	0.13 ± f	0.000 ± a	0.13	0.036
Ga01.22	1.23 ± e	0.23 ± e	0.002 ± b	0.23	0.044	1.15 ± g	0.15 ± g	0.001 ± b	0.15	0.039
Ga01.06	1.23 ± e	0.23 ± e	0.005 ± b	0.23	0.048	1.17 ± g	0.17 ± g	0.004 ± b	0.17	0.044
Ga01.08	1.20 ± d	0.20 ± d	0.001 ± a	0.20	0.045	1.13 ± f	0.13 ± f	0.001 ± b	0.13	0.039
Ga01.20	1.20 ± d	0.20 ± d	0.001 ± a	0.20	0.040	1.12 ± f	0.12 ± f	0.001 ± b	0.12	0.036
Ga02.01	1.23 ± e	0.23 ± e	0.003 ± b	0.23	0.046	1.19 ± g	0.19 ± g	0.003 ± b	0.19	0.042
Ga02.03	1.22 ± e	0.22 ± e	0.004 ± b	0.22	0.045	1.15 ± g	0.15 ± g	0.001 ± b	0.15	0.039
Ga02.07	1.24 ± e	0.24 ± e	0.004 ± b	0.24	0.048	1.17 ± g	0.17 ± g	0.003 ± b	0.17	0.043
Ga01.01	1.26 ± e	0.26 ± e	0.003 ± b	0.26	0.050	1.19 ± g	0.19 ± g	0.003 ± b	0.19	0.045
Ga01.02	1.24 ± e	0.24 ± e	0.004 ± b	0.24	0.048	1.16 ± g	0.16 ± g	0.002 ± b	0.16	0.042
Ga04.11	1.23 ± e	0.23 ± e	0.001 ± a	0.23	0.047	1.16 ± g	0.16 ± g	0.001 ± b	0.16	0.042
Ga02.02	na	na	na	na	na	1.19 ± h	0.19 ± h	0.002 ± b	0.19	0.045
Ga02.06	1.26 ± e	0.26 ± e	0.005 ± b	0.26	0.051	1.18 ± g	0.18 ± g	0.002 ± b	0.18	0.046
Ga04.08	1.24 ± e	0.24 ± e	0.002 ± b	0.24	0.046	1.17 ± g	0.17 ± g	0.002 ± b	0.17	0.040
Ga06.01	1.25 ± e	0.25 ± e	0.008 ± b	0.25	0.050	1.18 ± g	0.18 ± g	0.003 ± b	0.18	0.042
Ga06.02	1.27 ± e	0.27 ± e	0.012 ± b	0.27	0.053	1.21 ± h	0.21 ± h	0.004 ± b	0.21	0.048
Ga09.04	1.25 ± e	0.25 ± e	0.007 ± b	0.25	0.050	1.18 ± g	0.18 ± g	0.004 ± b	0.18	0.045
Ga07.01	1.26 ± e	0.26 ± e	0.005 ± b	0.26	0.051	1.19 ± g	0.19 ± g	0.002 ± b	0.19	0.047
Ga08.01	1.27 ± e	0.27 ± e	0.010 ± b	0.27	0.053	1.21 ± h	0.21 ± h	0.004 ± c	0.21	0.049
Ga09.03	1.26 ± e	0.26 ± e	0.014 ± c	0.26	0.053	1.18 ± g	0.18 ± g	0.008 ± c	0.18	0.046
Ga08.03	1.27 ± e	0.27 ± e	0.008 ± b	0.27	0.052	1.21 ± h	0.21 ± h	0.008 ± c	0.21	0.050
Ga10.02	1.25 ± e	0.25 ± e	0.000 ± a	0.25	0.049	1.18 ± g	0.18 ± g	0.004 ± b	0.18	0.042
Ga10.06	1.27 ± e	0.27 ± e	0.000 ± a	0.27	0.055	1.20 ± h	0.20 ± h	0.007 ± c	0.20	0.051
Ga08.05	1.28 ± e	0.28 ± e	0.007 ± b	0.28	0.054	1.23 ± h	0.23 ± h	0.003 ± b	0.23	0.049
Ga09.02	1.25 ± e	0.25 ± e	0.005 ± b	0.25	0.048	1.17 ± g	0.17 ± g	0.002 ± b	0.17	0.042
Ga10.08	1.24 ± e	0.24 ± e	0.000 ± a	0.24	0.046	1.18 ± g	0.18 ± g	0.001 ± b	0.18	0.042
Ga101B.3	1.25 ± e	0.25 ± e	0.000 ± a	0.25	0.050	1.18 ± g	0.18 ± g	0.002 ± b	0.18	0.045
Ga101B.5	1.26 ± e	0.26 ± e	0.000 ± a	0.26	0.052	1.19 ± h	0.19 ± h	0.001 ± b	0.19	0.046
Ga101B.m	na	na	na	na	na	1.18 ± g	0.18 ± g	0.002 ± b	0.18	0.044
Mean	1.22 ± b	0.22 ± b	0.004 ± b	0.24	0.048	1.19 ± c	0.18 ± c	0.003 ± b	0.17	0.043

*± SE = standard error with a, b, c, d, e, f, g, and h equal to 0, 0.001, 0.002, 0.005, 0.006, 0.008, 0.009, and 0.01; respectively.*

*na = Not applicable.*

*The Pearson correlation coefficient between the two groups for NA, Ho and %PL was 0.94 (P < 0.001); for NPA was 0.59 (P = 0.001), and for GD was 0.95 (P < 0.001).*

**FIGURE 4:**
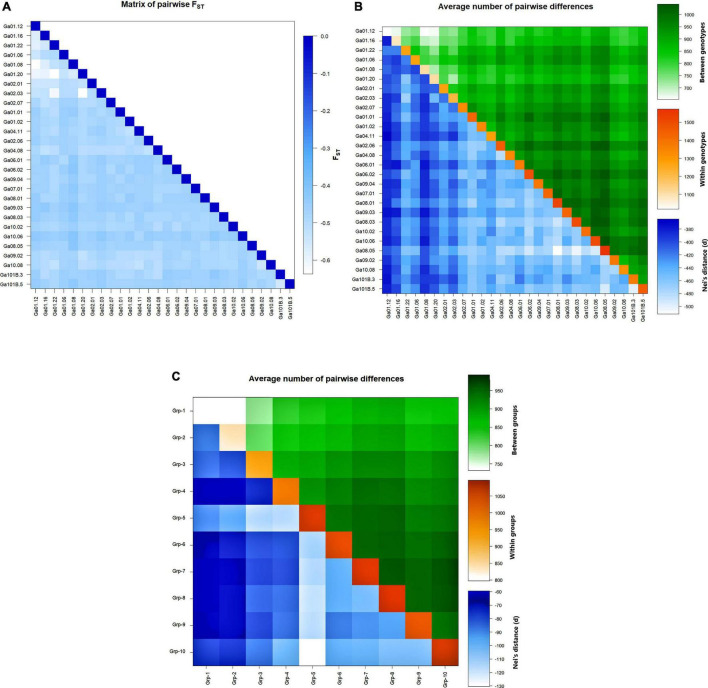
Heatmaps of **(A)** pairwise F_ST_ between the 28 genotypes, **(B)** average number of pairwise differences (above diagonal) and Nei’s distance (below diagonal) between the 28 genotypes, and average number of pairwise differences within the 28 genotypes (diagonal), and **(C)** average number of pairwise differences (above diagonal) and Nei’s distance (below diagonal) between the 10 groups of genotypes, and average number of pairwise differences within the 10 group of genotypes (diagonal).

Genotype Ga01.12 and Ga01.08 had a relatively low Nei’s distance and mean number of pairwise differences from most of the other genotypes, whereas genotype Ga08.05 had relatively high values in these parameters. The lowest mean number of pairwise differences between genotypes was recorded for Ga01.08 vs Ga01.12 and Ga01.20 vs Ga01.12. The lowest mean number of pairwise differences within genotype was observed in Ga01.12, followed by Ga01.16, Ga01.08, and Ga01.20. In contrast, the mean number of pairwise differences recorded for Ga08.05, Ga08.03, and Ga08.01 was among the highest (Figure 4). At a group level, Group-1 had the lowest mean number of pairwise differences within group whereas Group-10 had the highest. Group-1 vs Group-2 had the lowest mean number of pairwise differences between groups, while Group-6 vs Group-10 had the highest Nei’s distance (Figure 4C).

### Cluster, Principal Coordinate and Population Structure Analyses

**FIGURE 5:**
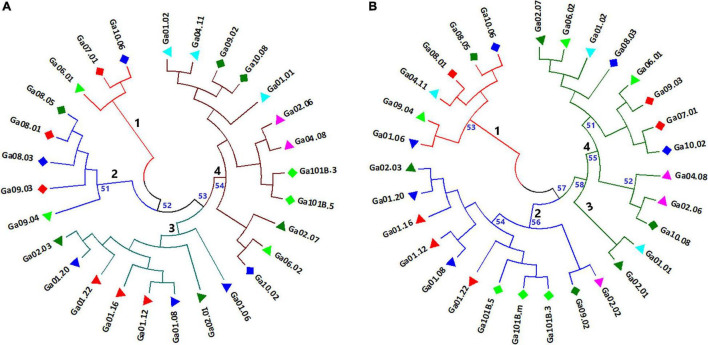
Neighbor-joining trees depicting the clustering pattern of **(A)** 28 noug genotypes and **(B)** 30 noug genotypes based on Nei’s standard genetic distances calculated using genotypic data at 5,531 and 1,687 SNP loci, respectively. The numbers in black represent the four clusters of each tree, and the numbers in blue represent the bootstrap support of the corresponding branches (only bootstrap values above 50 are shown).

**FIGURE 6:**
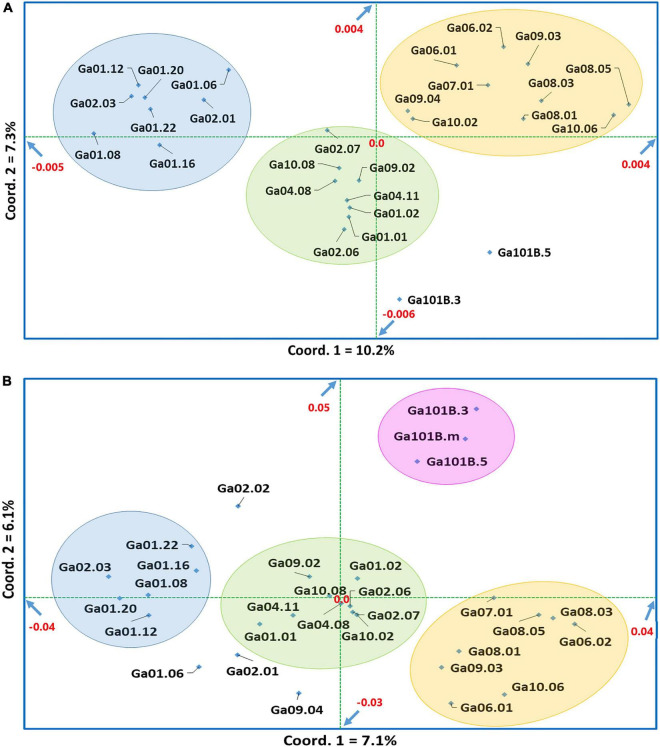
Principal coordinate analysis (PCoA) of **(A)** 28 noug genotypes and **(B)** 30 noug genotypes based on Nei’s standard genetic distances calculated using genotypic data at 5,531 and 1,687 SNP loci, respectively. The first two principal coordinates together explained 17.5 and 13.2% of the total variation in the case of panels **(A,B)**, respectively.

Neighbor-joining (NJ) cluster analysis and principal coordinate analysis (PCoA) were conducted based on Nei’s standard genetic distance (Supplementary Table 5) calculated using 5,531 and 1,687 SNP data sets for the 28 and 30 noug genotypes, respectively. Following the approach described in Brown-Guedira et al. (2000) for finding an acceptable number of clusters where the within-cluster genetic distance is below the overall mean genetic distance and where the mean between-cluster distance is above the mean within-cluster distance, four major clusters were determined in both cases. Even though there are clear differences between the clustering patterns generated with the two data sets, similarities are also evident. In both cases, genotype Ga10.06, Ga08.01, Ga08.05 and Ga09.04, which were assigned to cluster-1 or cluster-2, were among the most differentiated. On the other hand, genotypes that are less sensitive to photoperiod (Ga101B.3 and Ga101B.5) were closely clustered together in cluster-4 (Figure 5A) and cluster-2 (Figure 5B), respectively. Among the self-compatible genotypes, Ga01-12, Ga01-16 and Ga01-22 (red triangle) were closely clustered in cluster-3 (Figure 5A) and Cluster-2 (Figure 5B). In both cases, cluster-4 is the most diverse, comprising genotypes from seven of the ten groups (see symbols). In several cases, genotypes within the same phenotypic group were assigned to more than one clusters. For example, both very early-maturing genotypes (blue diamond) and very late-maturing genotypes (red diamond) were placed under more than one cluster (Figures 6A,B).

The principal coordinate analysis (PCoA) was conducted to determine the differentiation among the 28 individual genotypes (Figure 6A) and the 30 individual genotypes (Figure 6B), respectively. In the two-dimensional plots generated, the first and the second coordinates explained 10.2 and 7.3% of the total variation among the 28 individual genotypes (Figure 6A) and 7.1 and 6.1% of the total variation among the 30 individual genotypes (Figure 6B), respectively. Hence, the two coordinates together explained 17.5% of the total variation among the 28 individual genotypes and 13.2% of the total variation among the 30 individual genotypes, both of which are quite low. However, the clustering pattern of the genotypes in both two-dimensional plots (Figures 6A,B) are in good agreement, as clusters highlighted by the same color mostly represent the same genotypes. Most self-compatible genotypes (see Supplementary Table 1) were assigned to the light-blue highlighted clusters. The results of PCoA and cluster analysis also agree well in general. For example, similar to cluster analysis, genotypes less sensitive to photoperiod were closely clustered in PCoA (pink highlighted genotypes in Figure 6B). Analyses of the population genetic structure based on admixture models using 5,531 SNPs for the 28 genotypes and 1,687 SNPs for the 30 genotypes demonstrated that the genotypes are best represented by three genetic populations. (*K* = 3; Supplementary Figures 1A,C). It is interesting to note that each genotype has alleles originating from the three genetic populations, in both cases, demonstrating a strong genetic admixture (Supplementary Figures 1B,D).

## Discussion

### The SSR Characteristics in Noug Unigenes

The RNA-Seq based sequencing of 30 noug genotypes resulted in 409,309 unigenes ranging in size from 201 to 13,568 bp, totaling 204.2 Mbp, and having a G+C content of 40%. The G+C content is an important feature of genome organization, and show wide variation among different genomes and different regions within a genome, and has been studied in connection with understanding genome evolution (Morgante et al., 2002; Glémin et al., 2014; Singh et al., 2016). Diversity in G+C content in plant genomes is biologically and evolutionary significant, including its importance for plant adaptation to diverse environments (Šmarda et al., 2014). Studies have shown that grasses, such as rice and maize, have genomic G+C content above 40%, while dicots have G+C content below 40% (Wang et al., 2004; Qin et al., 2015; Singh et al., 2016). In general, genes have a higher G+C content than genomic sequences, with their coding sequences (CDS) having a higher G+C content than their 3′ and 5′ untranslated regions (3′-UTR and 5′-UTR) (Zhao et al., 2014; Singh et al., 2016). The G+C content of CDS exceeds 40% even for dicots (Wang et al., 2004; Singh et al., 2016). Hence, the G+C content of 40% obtained in the present study for the noug unigenes (CDS plus UTRs) is consistent with data reported for other dicots.

Simple sequence repeats (SSRs) are ubiquitous and highly polymorphic loci in plant genomes comprising tandemly repeated nucleotide sequences of 1 to 6 bp in length. Genomic events that lead to the length polymorphism of SSRs include unequal recombination between homologous SSRs and replication slippage that result in repeat motif deletion or insertion (Li et al., 2004). In the CDS, frameshift mutations that result in a gain or loss of function occur as the result of insertions or deletions of the SSR repeat motifs (Li et al., 2004). The high mutation rate of SSRs makes them a significant component of genome evolution (Kashi et al., 1997), and they are excellent molecular markers for various applications (Olmstead et al., 2008; Geleta et al., 2012; Lu et al., 2012; Shiferaw et al., 2012; Shirasawa et al., 2013; Teshome et al., 2015; Chombe et al., 2017). The distribution and density of SSRs vary among genomes of different species as well as different regions within genomes (Tóth et al., 2000; Temnykh et al., 2001; Mun et al., 2006). Similarly, the frequency of different types of SSRs (mono, di, tri, tetra, penta, and hexanucleotide repeats) as well as the nucleotide composition of their repeat motifs differ within and among genomes (Morgante et al., 2002; Grover and Sharma, 2007; Qin et al., 2015).

Mononucleotide repeats were the most frequent in the present study accounting for over half of the SSRs identified, of which A/T SSRs accounted for 97.2%. Similarly, AT/TA SSRs were by far more prevalent among dinucleotide repeat SSRs than CG/GC SSRs, accounting for 32.4 and 0.24% of all dinucleotide repeat SSRs, respectively. Such an overwhelming dominance of A/T over C/G and AT/TA over CG/GC in noug unigenes is consistent with that of mono and dinucleotide repeat SSRs in the genomes of other dicots, including *Arabidopsis thaliana*, *Glycine max*, *Vitis vinifera* and *Solanum lycopersicum* (Qin et al., 2015). According to Qin et al. (2015), C/G and CG/GC SSRs declined during the evolution of plant genomes, which warrants further research to identify the major causes for this change. In other groups of dinucleotide repeat SSRs, homopurine/homoprymidine motifs (AG/CT+GA/TC) were more frequent than purine-prymidine mix (AC/GT+CA/TG) in the present study, in agreement with other studies in dicots (Grover and Sharma, 2007; Wang et al., 2008; Qin et al., 2015). The relative frequency of tri, tetra, penta, and hexanucleotide SSR motifs differed among studies, even within dicots, in contrast to mono and dinucleotide repeat SSRs. Trinucleotide repeat SSRs are more common than dinucleotide repeat SSRs in Arabidopsis CDS and UTRs (Morgante et al., 2002), unlike the case in the present study, where they are more or less equally frequent. The two most common trinucleotide SSRs in the present study were those with ACC/GGT and CCA/TGG motifs, unlike in Papaya where the AAG motif dominates the trinucleotide SSRs (Wang et al., 2008). Among complimentary motifs, notable differences exist between GGT and ACC, and between GAA and TTC, accounting for 6.4, 3.7, 4.0, and 2.2% of trinucleotide repeat SSRs, respectively. Hence, higher frequencies of GGT and GAA in the transcribed sequences of noug require further research in comparison with other dicots.

The G+C content in the noug unigenes (40%) is significantly higher than the G+C content of the SSRs derived from these unigenes (22.2%). Similar pattern was reported in *Populus* where 33.2% G+C content in the whole genome but 25.4% in the SSRs (ShuXian and TongMing, 2007). The CCG/CGG trinucleotide repeats are abundant in monocots (rice, maize, and wheat) but rare in dicots (Arabidopsis and soybean) (Morgante et al., 2002). They are among low-frequency trinucleotide SSRs in the present study, which is similar to the results from Morgante et al. (2002) study for dicots. Also, they found higher G+C content in monocot ESTs than in dicot ESTs. Nevertheless, the G+C content of 44% in EST-derived Arabidopsis and soybean SSRs they reported is twice that of the present study’s noug SSRs (22%). The marked difference between the two studies could be partly attributed to differences in the representation of CDS and UTRs in the respective sequences; further studies will shed more light on this. The higher G+C content of trinucleotide SSRs than di and tetraploid SSRs in the present study is most likely due to the greater number of GC-rich trinucleotide SSRs in CDS, which do not cause frameshift mutations.

Using transcriptome-based SSR markers, previous studies on noug revealed higher genetic variation both within and between populations (Dempewolf et al., 2010, 2015; Misganaw and Abera, 2017) in comparison to results obtained using dominant markers (Geleta et al., 2007, 2008) and bi-allelic SNP markers (Tsehay et al., 2020), indicating the superiority of multi-allelic SSR markers in their informativeness. Since a reference genome sequence is not available for noug yet, the genomic positions of the SSRs identified in the present study is currently unknown. With the annotation of the unigenes used, it will be possible to select genome-wide single-copy SSRs for genotyping a diverse panel of noug genotypes and then develop gene-associated polymorphic SSRs for their numerous applications in noug and other *Guizotia* species, as the rate of cross-species transferability of transcriptome-derived SSR markers is proved high (Lu et al., 2013; Teshome et al., 2015; Zhou et al., 2016; Gadissa et al., 2018; Serbessa Tolera et al., 2021). The applications include genetic diversity analyses for conservation and breeding, as well as genetic linkage mapping and genome-wide association studies.

### The SNP Markers and Genetic Variation Among Genotypes

Given that noug is strictly outcrossing in general (Nemomissa et al., 1999; Geleta and Bryngelsson, 2010; Geleta and Ortiz, 2013), observed heterozygosity (Ho) is expected to equal or exceed the expected heterozygosity (He) if other HWE assumptions are met. However, a very small fraction of the SNP loci (< 0.5%) exhibited heterozygote deficiency. Hence, natural selection may be favoring homozygosity at these loci although self-pollination might have contributed to the heterozygote deficiency given that 40% of the genotypes are self-compatible to different extents. Contrary to this, 9.9% of the loci (Supplementary Table 4) were heterozygous across all genotypes, which is particularly interesting when considering loci with proportional allele frequencies. Natural selection favoring heterozygosity might have contributed, but genotype calling based on reads from duplicate genes with different alleles cannot be ruled out. The development of a reference genome sequence for noug, as well as the annotation and comparison of the unigenes with sunflower genes (the closest to noug among well-studied crops) will provide evidence that explain these results.

As the number of markers increased from 1,687 (Ho = 0.18) to 5,531 (Ho = 0.22), the mean observed heterozygosity (Ho) also increased, suggesting that the number of markers influences the parameter, particularly for small number of samples. Whereas, a study on 24 noug accessions comprising 281 genotypes reported a slightly higher Ho (0.24) based on 202 transcriptome derived-polymorphic SNP markers (Tsehay et al., 2020), suggesting a stronger effect of sample size than number of markers. Considering the analysis of 28 genotypes using 5,531 markers, Ho varied from 0.18 (Ga01.12) to 0.28 (Ga08.05). On average, self-compatible genotypes were less heterozygous than their self-incompatible counterparts were, and the lower Ho values in self-compatible genotypes resulted from self-pollination. There is, however, still substantial heterozygosity in self-compatible genotypes that have been self-pollinated for a number of generations. As inbreeding depression in noug is high (Geleta and Bryngelsson, 2010; Geleta and Ortiz, 2013), a significant proportion of plants grow poorly following self-pollination. As a result, selecting plants with higher proportions of heterozygous loci for next round breeding is more likely, explaining the high heterozygosity of the self-compatible genotypes. Consequently, developing pure-line cultivars is likely to be challenging although self-compatible genotypes were successfully developed.

Polymorphism information content (PIC) measures the usefulness of DNA markers in terms of detecting genetic variation (Hildebrand et al., 1992; Shete et al., 2000). The PIC of a locus depends on the number and frequency of its alleles, which in turn depends on the diversity of genotypes (populations) analyzed. In the larger data set 5,531 SNPs, 19.4% had a PIC of above 0.30, which makes them highly informative. In a similar study in noug, 50% of the 202 markers used had a PIC value above 0.25 (Tsehay et al., 2020). Comparatively, 31% of polymorphic markers had PIC values above 0.25 in this study. The lower proportion in this study can be explained by a smaller number of samples used compared to Tsehay et al. (2020). Nevertheless, 1,266 SNP markers had PIC exceeding 0.25 (1,074 of which had PIC above 0.30; Supplementary Table 3), which can be prioritized for use in various applications, including population genetics for conservation and breeding, genetic linkage mapping, and genome-wide association studies.

There is a good correlation between results obtained from the analyses of the data sets containing 5,531 and 1,687 SNPs, although the values of most parameters analyzed are higher for the larger data set. In both cases, the highest mean genetic distance was recorded in genotype Ga10.06 and the highest number of private alleles was recorded in genotype Ga09.03. Both genotypes are self-incompatible but they mature at different times. The genotype Ga10.06 was sampled from a very early-type landrace population that was originally collected from Arsi (39 km from Bekoji to Tereta; southeast Ethiopia), whereas the genotype Ga09.03 was sampled from a very late-type population that was originally collected from Gojjam (35 km from Amanuel to Bure; northwest Ethiopia). A higher mean genetic distance of Ga10.06 is not surprising since it came from an isolated location where the cultivation of noug is low. Ga09.03 was sampled from a major noug growing region that it shared with the other two very late-type genotypes (Ga07.01 and Ga08.01), so its relatively high number of private alleles was noteworthy given the high rate of gene flow within the region (Geleta et al., 2008).

The lowest mean number of pairwise differences (MNPD) among genotypes were recorded between pairs of self-compatible genotypes (Ga01.08 vs Ga01.12 and Ga01.20 vs Ga01.12). Self-compatible genotypes are developed through crossbreeding of a few genotypes that exhibit a low level of self-compatibility, and hence, their low pairwise differences is due to their narrow genetic basis. The lowest mean number of pairwise differences within genotypes (e.g., heterozygosity) was also recorded in self-compatible genotypes, which is not surprising since the genotypes have been self-pollinated for a number of generations, and hence increased homozygosity as compared to the self-incompatible genotypes. Those that exhibited the highest mean number of pairwise differences within genotypes (Ga08.05, Ga08.03, and Ga08.01) are all strictly self-incompatible.

### Genetic Variation of Genotypes Within Trait-Based Groups

The 30 noug genotypes used in the present study were grouped into 10 different groups based on their phenotypic characteristics. Each group differs from the others at least in one characteristic in terms of ability to set self-seeds, photoperiod sensitivity, duration to reach seed maturity, and seed oil and oleic acid contents. However, the genotypes within each group were genetically diverse with the exception of Group-1 (Ga01.12, Ga01.16, and Ga01.22) comprising genotypes bred for higher oil content, and Group-10 (Ga101B.3, Ga101B.5, and Ga101B.m) comprising genotypes with a lower photoperiod sensitivity (Supplementary Table 1).

Overall, the self-compatible groups were more closely related to one another than the self-incompatible ones. The self-compatible genotypes were developed through crossbreeding and selfing based on a limited number of genotypes originating from a few landrace populations. As such, their relatively higher genetic relationship is a result of their narrow genetic base and the crossbreeding scheme used. Interestingly, both the cluster analysis and principal coordinate analysis assigned genotypes with oil content above 40% to more than one cluster. For example, Ga01.20 and Ga02.07 are both high oil content genotypes (over 40%) and self-compatible genotypes (Ga01.20 being among the best for self-compatibility) but they were assigned to different clusters in both analyses. Hence through crossbreeding these genotypes, a self and cross-pollinating cultivar with high seed and oil yields can be developed. It would be very interesting to apply such an approach to noug, as it can overcome the potential consequences of inbreeding depression.

The dominant fatty acid in noug seed oil is linoleic acid (C18:2) and oleic acid (C18:1) content is generally below 13%, particularly in noug grown in Ethiopia (Dagne and Johnson, 1997; Geleta et al., 2011; Tsehay et al., 2020). However, genotypes with C18:1 above 13% have been identified and crossbred to develop high oleic acid types (Geleta et al., 2011; Geleta and Ortiz, 2013) although their oleic acid levels fluctuate with the average temperature of the growing environments. They produce significantly higher C18:1 at the expense of C18:2 in low-altitudes [below 1,800 meters above sea level (masl)] than in high-altitudes (above 2,200 masl) (Geleta et al., 2011; Tsehay et al., 2020). Among the genotypes included in the present study, three self-compatible genotypes (Ga01.16, Ga02.01, and Ga01.02) and one self-incompatible genotype (Ga02.02) had an oleic acid content above 13%, except when grown in high-altitude environments. The data analyses revealed that these genotypes are genetically diverse and differ in desirable traits, such as oil content. Therefore, their crossbreeding may result in high-oleic acid noug cultivars suitable for low-altitude cultivation. Early maturity is a highly desirable trait in crops, especially when the growing season is short or in drought-prone areas, but it usually comes at a cost in terms of yield (Cattivelli et al., 2008). The genotypes included, in the present study varied from “very-early” type to “very-late” type, which took ca 120 and 180 days from planting to harvesting, respectively, when grown at a high-altitude location (Holeta agricultural research center in Ethiopia; 9^°^00′ N, 38°30′ E; 2400 masl). Based on pairwise comparison as well as cluster and principal coordinate analyses, Group-8 (Ga08.03, Ga10.02, and Ga10.06) consisted of very-early type self-incompatible genotypes, which are genetically diverse. Crossbreeding these genotypes can therefore improve various desirable traits without affecting their earliness.

Research in population genetics uses various approaches to determine the genetic structure of populations and the source of genotypes (Rannala and Mountain, 1997; Davies et al., 1999; Pritchard et al., 2000; Alexander et al., 2009; Raj et al., 2014). In the present study, a model-based approach of Pritchard et al. (2000) was used for population structure analysis, which assumes that populations are characterized by a set of allele frequencies across multiple loci. By using this approach, each individual within a predefined population is probabilistically assigned to a cluster, or it is assigned to multiple clusters if it is determined to be admixed. The genotypes in the present study were analyzed to determine the population genetic structure using this model. The analysis using the Puechmaille (2016) approach determined that the optimal number of clusters (K) is three, corresponding to three genetic populations. Interestingly, all genotypes are the results of admixture from the three genetic populations with a slightly different extent. This significant level of admixture may have caused the discrepancy between the four clusters obtained from cluster analysis and PCoA compared to the three clusters obtained from Bayesian statistics-based population genetic structure analysis. A recent study on 24 diverse noug accessions comprising 281 genotypes also revealed three genetic populations with strong admixture (Tsehay et al., 2020). The studies generally suggest a weak population structure in noug due to population admixture caused by strong gene flow between populations *via* pollen and germplasm exchange that gradually covers wide geographic areas.

## Conclusion

Through RNA-Seq based sequencing, 409,309 unigenes, representing the noug transcriptome, have been developed for its various applications in the present study. The G+C content of these unigenes was 40%, which is comparable to that of other dicots. The analyses of SSRs in the unigenes revealed an overwhelming predominance of A/T over C/G and AT/TA over CG/GC, consistent with other dicots. Interestingly, GGT and GAA repeats had a higher frequency than their complementary motifs. This suggests their greater importance in noug genes, and therefore requires further investigation in comparison with other dicots. The whole unigenes are significantly higher in G+C content (40%) than the SSRs derived from them (22.2%). Further research and analysis of the SSRs identified in the current study could lead to the development of genome-wide single-copy SSRs with high polymorphism for use in noug breeding and research. Thousands of high-quality SNPs were discovered in each noug genotype in the present study, and well over a thousand of them were common to all genotypes and possessed a high polymorphism information content (PIC > 0.30), which makes them ideal for use in a wide range of applications. The significant levels of admixture observed in each noug genotypes suggest a weak population structure in noug likely caused by strong gene flow between populations across wide geographic areas. Although the self-compatible genotypes were bred for several generations with self-pollination, a substantial level of heterozygosity was observed, suggesting an inbreeding depression that led to plants with higher heterozygosity being selected in successive generations, presenting potential challenges to the development of highly productive and nutritionally rich pure-line cultivars. Interestingly, genotypes that share desirable characteristics, such as self-compatibility, early maturity, high oil content, or high oleic acid content are genetically diverse. Crossbreeding these genotypes would enable the development of cultivars that combine these characteristics and reproduce through both selfing and cross-pollination, which would be a viable approach to overcome the potential effects of inbreeding depression.

## Data Availability Statement

The datasets presented in this study can be found in online repositories. The names of the repository/repositories and accession number(s) can be found below: National Center for Biotechnology Information (NCBI) BioProject database under accession numbers GJSF00000000 and PRJNA763316 (https://www.ncbi.nlm.nih.gov/bioproject/PRJNA763316).

## Author Contributions

MG and RO: conceptualization. AG, MG, RV, and CH: methodology. AG and MG: software. AG, MG, CH, and RO: data analysis. AG: writing–original draft. RO, MG, and KT: funding acquisition. All authors have contributed in supervision, writing—review and editing, read and agreed to the published version of the manuscript.

## Conflict of Interest

The authors declare that the research was conducted in the absence of any commercial or financial relationships that could be construed as a potential conflict of interest.

## Publisher’s Note

All claims expressed in this article are solely those of the authors and do not necessarily represent those of their affiliated organizations, or those of the publisher, the editors and the reviewers. Any product that may be evaluated in this article, or claim that may be made by its manufacturer, is not guaranteed or endorsed by the publisher.

## Footnotes

1
http://pgrc.ipk-gatersleben.de/misa/misa.html

## Supplementary Material

Supplementary Figure 1 Graphs depicting three clusters (*K* = 3) as the best representation of **(A)** the 28 noug genotypes based on 5,531 SNP loci, and **(C)** the 30 genotypes based on 1,687 SNP loci, using the method of Puechmaille (2016) for the determination of the optimum number of clusters; and the corresponding graphical display of the genetic structure of **(B)** 28 noug genotypes and **(D)** 30 noug genotypes generated based on genotypic data at 5,531 and 1,687 SNP loci, respectively, following the determination of the optimum number of clusters (K) of three (*K* = 3) using the method. The three colors in **(B)** and **(C)** correspond to the three clusters (genetic populations) and the proportion of each color in each genotype denotes the average proportion of the alleles that placed each accession under the three clusters.

Supplementary Table 1 Plant material (genotypes) used for this study and their general description.

Supplementary Table 2 The list of SSRs detected within the 35639 Unigenes and their descriptions.

Supplementary Table 3 List of the 5531 SNP loci that passed the quality filtering criteria and recorded accross 28 noug genotypes, together with their SNP position (POS), reference allele (REF), alternative allele(s) (ALT), quality score, (QUAL), polymorphic information content (PIC), Observed heterozygosity (Ho), expected heterozygosity (He), *P*-value for Hardy-Weinbeg Equlaibrium (HWE *P*-value) and corrsponding reference unigene sequence.

Supplementary Table 4 List of SNP loci that showed signfcant deviation from Hardy-Weinberg Equlibrium (HWE), under the assumption that the 28 genotypes represent a single population, together with their SNP position (POS), reference allele (REF), alternative allele(s) (ALT), Observed heterozygosity (Ho), expected heterozygosity (He), Ho-He and *P*-value for Hardy-Weinbeg Equlaibrium (HWE *P*-value).

Supplementary Table 5 The pairwise Nei’s standard genetic distance between the 28 noug genotypes calculated based on 5531 SNP loci. The diagonal values are mean genetic distance of each genotype from all other genotypes. The Pearson correlation coefficent between the mean genetic distance for 28 genotypes (5531 polymorphic loci) and the 30 genotypes (1687 polymorphic loci) was 0.955 (*P* < 0.00001).
