# Supplementary figures and images for: RNA-Seq Provides Novel Genomic Resources for Noug (Guizotia abyssinica) and Reveals Microsatellite Frequency and Distribution in Its Transcriptome
Source: Front Plant Sci. 2022 May 11;13:882136. doi: 10.3389/fpls.2022.882136 (PMC9132581; doi:10.3389/fpls.2022.882136)

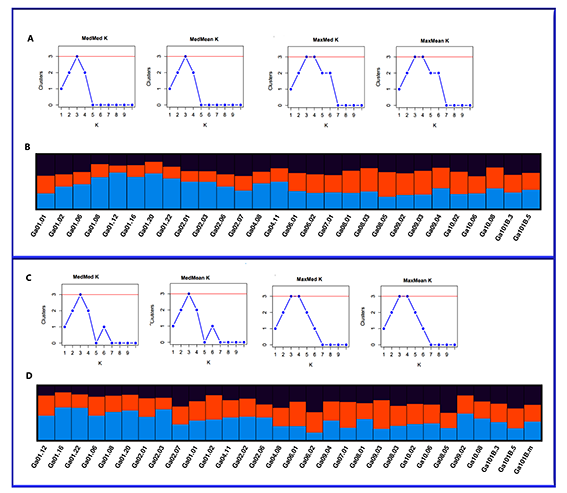

Supplement: Supplementary Figure 1 — Graphs depicting three clusters (K = 3) as the best representation of (A) the 28 noug genotypes based on 5,531 SNP loci, and (C) the 30 genotypes based on 1,687 SNP loci, using the method of Puechmaille (2016) for the determination of the optimum number of clusters; and the corresponding graphical display of the genetic structure of (B) 28 noug genotypes and (D) 30 noug genotypes generated based on genotypic data at 5,531 and 1,687 SNP loci, respectively, following the determination of the optimum number of clusters (K) of three (K = 3) using the method. The three colors in (B) and (C) correspond to the three clusters (genetic populations) and the proportion of each color in each genotype denotes the average proportion of the alleles that placed each accession under the three clusters. [file Image_1.tiff]
